# Continuous treatment with FTS confers resistance to apoptosis and affects autophagy

**DOI:** 10.1371/journal.pone.0171351

**Published:** 2017-02-02

**Authors:** Eran Schmukler, Eya Wolfson, Zvulun Elazar, Yoel Kloog, Ronit Pinkas-Kramarski

**Affiliations:** 1 Department of Neurobiology. Tel-Aviv University, Ramat-Aviv, Israel; 2 Department of Biological Chemistry; The Weizmann Institute of Science; Rehovot, Israel; Univerzitet u Beogradu, SERBIA

## Abstract

High percentage of human cancers involves alteration or mutation in Ras proteins, including the most aggressive malignancies, such as lung, colon and pancreatic cancers. FTS (Salirasib) is a farnesylcysteine mimetic, which acts as a functional Ras inhibitor, and was shown to exert anti-tumorigenic effects *in vitro* and *in vivo*. Previously, we have demonstrated that short-term treatment with FTS also induces protective autophagy in several cancer cell lines. Drug resistance is frequently observed in cancer cells exposed to prolonged treatment, and is considered a major cause for therapy inefficiency. Therefore, in the present study, we examined the effect of a prolonged treatment with FTS on drug resistance of HCT-116 human colon cancer cells, and the involvement of autophagy in this process. We found that cells grown in the presence of FTS for 6 months have become resistant to FTS-induced cell growth inhibition and cell death. Furthermore, we discovered that the resistant cells exhibit altered autophagy, reduced apoptosis and changes in Ras-related signaling pathways following treatment with FTS. Moreover we found that while FTS induces an apoptosis-related cleavage of p62, the FTS-resistant cells were more resistant to apoptosis and p62 cleavage.

## Introduction

The Ras family of small GTPases consists of 4 highly related members: K-Ras (4A and 4B), H-Ras and N-Ras [1]. Ras proteins are typically found in the inner leaflet of the plasma membrane, where their activation leads to signal transduction through interaction with multiple effector proteins. Activation of Ras guanine nucleotide exchange factors (RasGEFs) results in the exchange of the Ras bound GDP into GTP. Following activation, Ras stimulates diverse downstream effectors, which lead to the activation of an array of cellular signaling pathways. Activating mutations in Ras are found in 33% of human cancers, with mutations in K-Ras being the most prevalent (21.6% of human cancers) [2]. K-Ras mutations are associated mainly with the most lethal malignancies, such as lung, colon and pancreatic cancers [3]. While the wild-type (wt) Ras cycles between its active and inactive states, and is regulated by RasGEFs and RasGAP (GTPase), the oncogenic mutant Ras, which bears a single mutation at residues 12, 13 or 61, binds to GTP constitutively [1].

Activation of Ras requires its association with the cell membrane. This characteristic of Ras is used to design anti-Ras drugs that inhibit its association with the plasma membrane [4]. Several such agents, which have the potential of inhibiting Ras by mimicking its farnesyl group, were developed. One of these agents, FTS (farnesylthiosalicylic acid, also known as Salirasib), acts as a functional Ras antagonist, and was shown to exert anti-tumorigenic effects *in vitro* and *in vivo* [5–8]. FTS affects Ras-membrane interactions by dislodging Ras from the membrane anchoring domains, thus facilitating its degradation [9]. FTS treatment was shown to induce autophagy in naïve mouse embryonic fibroblasts (MEF) and in human cancer cell lines, which harbor a K-Ras mutation (HCT-116, DLD-1 and Panc-1) [10,11]. Autophagy is a regulated process, by which proteins and organelles are recognized and delivered to the lysosome for degradation [12]. FTS-induced autophagy acts as a defense mechanism against FTS-induced cell death [10,11]. In addition, FTS enhances the synthesis of p62, which is essential for cargo selection during autophagy [11].

In the present study, we examined the effect of prolonged FTS treatment on cancer cells resistance to FTS-induced growth inhibition, cell death and autophagy. We found that HCT-116 human colon cancer cells treated with FTS for 6 months have become resistant to FTS treatment. Further characterization of these cells revealed changes in autophagy, p62 levels and cleavage, response to other anti-cancer treatments and activation of signaling pathways.

## Materials and Methods

### Antibodies and reagents

Antibodies are as follows: monoclonal mouse anti-actin (MP Biomedicals; Santa Ana, CA; 691001), polyclonal rabbit anti-caspase 3 (Santa Cruz Biotechnology; Dallas, TX; sc-7148 and Cell Signaling Technology; 9662), polyclonal rabbit anti-AKT (Santa Cruz Biotechnology; sc-8312), polyclonal rabbit anti-p21 (Santa Cruz Biotechnology; sc-756), polyclonal rabbit anti-p62 (MBL International; Woburn, MA; PM045), monoclonal rabbit anti-aurora kinase A (AURKA; Cell Signaling Technology; Denver, MA; 4718), polyclonal rabbit anti-ERK1/2 (Cell Signaling Technology; 4695), polyclonal rabbit anti-phospho-Ser473 AKT (Cell Signaling Technology; 4058), polyclonal rabbit anti-phospho-Thr389-S6 kinase (p-S6K; Sigma-Aldrich; St. Louis, MO; S6311), polyclonal rabbit anti-S6 kinase (S6K; Sigma-Aldrich; S4047), monoclonal mouse anti-phospho-Thr183 and Tyr185 ERK1/2 (Sigma-Aldrich; M8159) polyclonal rabbit anti-LC3B (Immunoblots; Sigma-Aldrich; L7543) and monoclonal rabbit anti-LC3A/B (Immunostaining; Cell Signaling Technology; 12741). FTS (SaliRasib, S-trans, trans-farnesylthiosalicylic acid) was provided by Concordia Pharmaceuticals (Fort Lauderdale, FL); chloroquine (CQ; C6628) and 5-fluorouracil (5-FU; F6627) were from Sigma-Aldrich; QVD-OPH was from R&D systems (Minneapolis, MN; OPH-001); calpeptin was from EMD Millipore (Darmstadt, Germany; 03-34-0051); and rapamycin was from Cayman Chemical (Ann Arbor, MI; 13346).

### Cell culture and generation of FTS-resistant sublines

To generate FTS-resistant HCT-116 sublines, naïve human colon cancer HCT-116 cells were grown in RPMI-1640 medium (Sigma-Aldrich) supplemented with 5% heat-inactivated fetal bovine serum (FBS; Hyclone, Thermo Scientific, Waltham, MA), containing FTS at a sub-IC50 concentration of 40 μM (prepared from a 75 mM in DMSO stock). FTS concentration was gradually increased during a period of 6 months up to a final concentration of 60 μM, and the cells were routinely passaged when confluence was achieved. Two sublines were simultaneously generated and designated FR1 (FTS-resistant1) and FR2 HCT-116. These sublines were continuously cultured in RPMI-1640 medium supplemented with 5% FBS, containing 60 μM FTS. Three days before each experiment, FTS was removed from the culture medium. The concentrations and the duration of FTS treatments (and the corresponding 0.1% DMSO control) are indicated for each experiment. An additional subline was generated from FR2 cells, which were further grown at increasing FTS concentrations. This subline was termed FR3, and was cultured at a final concentration of 72.5 μM FTS. A control HCT-116 subline was also generated by culturing naïve HCT-116 cells in RPMI-1640 medium supplemented with 5% FBS, containing 0.1% DMSO.

The human pancreatic cancer cell line, Panc-1, was grown in DMEM (Gibco, Carlsbad, CA), supplemented with 10% heat-inactivated fetal bovine serum (or 5% for FTS treatments).

### Assessment of cell viability and cell death

Cells were plated in medium supplemented with 5% FBS, and treated as indicated. Cell viability was determined by the methylene blue assay. The cells were fixed with 4% formaldehyde for 2 hours, then washed once with 0.1 M boric acid (pH 8.5) and incubated with the DNA-binding dye methylene blue (1% in boric acid) for 20 minutes at room temperature. The cells were then washed three times with distilled water and lysed with 0.1 M HCl. Absorbance was measured with a Tecan Spectrafluor Plus spectrophotometer (Mannedorf, Switzerland) at 595 nm. Cell viability is presented relative to untreated cultures. IC50 values were calculated using a non-linear regression model (logarithmic inhibitor vs. normalized response-variable slope) with the GraphPad Prism 6 software.

To estimate the number of dead cells, live cultures were incubated for 10 minutes with 1 μg/ml of the fluorescent, membrane impermeable, DNA dye bisbenzimide (Hoechst 33258; Sigma). After staining, the cells were photographed with an Olympus motorized inverted microscope Model IX81 (×20 magnification). The percentage of dead cells was estimated by calculating the number of Hoechst-stained nuclei relative to the total cell number in each field.

### Lysate preparation and immunoblot analysis

Cells were exposed to the indicated stimuli. After treatment, cells were lysed in solubilization buffer (50 mM HEPES pH 7.5, 150 mM NaCl, 10% glycerol, 1% Triton X-100, 1 mM EDTA pH 8, 1 mM EGTA pH 8, 1.5 mM MgCl_2_, 200 μM Na_3_VO_4_, 150 nM aprotinin, 1 μM leupeptin and 500 μM 4-(2-aminoethyl) benzenesulfonyl fluoride hydrochloride). Lysates were cleared by centrifugation, a sample buffer was added and the lysates were boiled. Lysates were resolved by SDS-polyacrylamide gel electrophoresis (PAGE) through 10%-12.5% polyacrylamide gels, and were electrophoretically transferred to nitrocellulose membranes. Membranes were blocked for 1 hour in TBST buffer (0.05 M Tris-HCl pH 7.5, 0.15 M NaCl, and 0.1% Tween 20) containing 6% milk, and then blotted with primary antibodies for 2 hours. Secondary antibody, linked to horseradish peroxidase, was then added for 1 hour. Immunoreactive bands were detected with the enhanced chemiluminescence reagent. Densitometric analysis of the results was performed using the ImageJ program.

### Immunostaining

For immunostaining of endogenous LC3, cells were plated on sterile coverslip (18 mm diameter) coated with poly-l lysine. After treatment, cells were fixed with cold methanol for 15 min and permeabilized/blocked with 2% normal goat serum in 0.3% Triton X-100 for 60 min. Cells were incubated with primary antibodies overnight at 4°C followed by staining with secondary antibodies and 2 μg/ml Hoecsht 33258 for 1 h at RT. Cells were analyzed by Leica TCS SP8 confocal microscope (×63 magnification). For the quantification of LC3 puncta, we used the ImageJ software to measure the total area of LC3 dots per cell.

### RNA extraction and qRT-PCR analysis

Total RNA was extracted from the cells using TRIzol reagent (Ambion Life Technologies, Grand Island, NY, USA) and the RNA concentration in each sample was determined by a spectrophotometer. Purified RNA was reverse-transcribed using the High Capacity cDNA Revese Transcription kit (Applied Biosystemns, Foster City, CA, USA) according to the manufacturer’s instructions. cDNA samples (50 ng) were used for quantitative RT–PCR using the FastStart Universal SYBR Green Master kit (Roche, *Basel*, Switzerland). Fluorescence was measured and the data were analyzed using the 7300 Real Time PCR system (Applied Biosystems). The primers used were human Aurora kinase A (AURKA) forward, TTCAGGACCTGTTAAGGCTACA and reverse, ATTTGAAGGACACAAGACCCG; human p62 forward, CAGAGAAGCCCATGGACAG and reverse, AGCTGCCTTGTACCCACATC; human GUSB forward, CTCATTTGGAATTTTGCCGATT and reverse, CCGAGTGAAGATCCCCTTTTTA. GUSB was used as a reference gene for normalization of relative mRNA expression.

### Transient transfections

Cells were transfected with 3 μg of either GFP-expressing or GFP-DN-Ras (17N) expressing vectors using the jetPEI reagent (Polyplus transfection, 101–10), according to the manufacturer's instructions.

### Statistical analysis

All experiments were performed at least three times. Results are presented as means ± SE. Differences between means were assessed by the 1-tailed Student’s t-test. Significance was assigned at *p* < 0.05.

## Results

### Generation of FTS-resistant HCT-116 sublines

**Fig 1:**
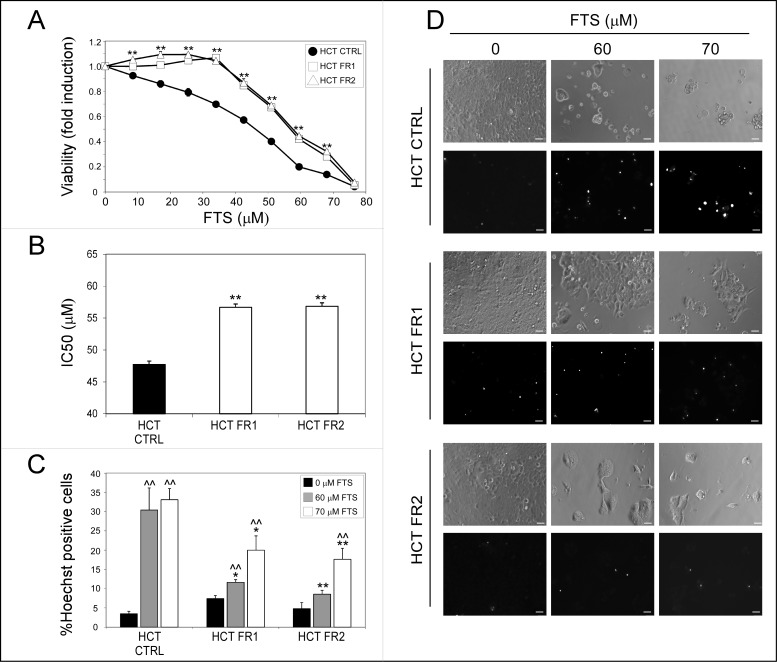
Continuous treatment with FTS induces resistance. CTRL, FR1 and FR2 HCT-116 cells were treated with increasing concentrations of FTS for 5 days. (A) cell viability was assessed using the methylene blue staining assay and (B) IC50 values were calculated as described in Materials and methods (mean ± SE; **, *p* < 0.01, CTRL compared to FR1 and FR2 HCT-116 cells). (C-D) CTRL, FR1 and FR2 HCT-116 cells treated with FTS at concentrations of 60 and 70 μM for 5 days were stained with the fluorescent DNA dye bisbenzimide (Hoechst 33258, 1 μg/ml) to assess the number of dying cells. Following staining, the cells were photographed (×20 magnification; scale bars, 20 micrometer). The percentage of dying cells was estimated by counting the number of Hoechst-positive cells compared to the number of total cells in each field (7–10 fields for each treatment, 100–200 cells per field). Results are the mean ± S.E (*, *p* < 0.05; **, *p* < 0.01, CTRL compared to FR1 and FR2 HCT-116 cells; ^^, *p* < 0.01, FTS treated compared to untreated cells).

To determine whether continuous treatment with FTS induces drug resistance in human HCT-116 colon cancer cells (expressing endogenous mutant K-Ras), the cells were grown in the presence of increasing FTS concentrations for 6 months, up to a final concentration of 60 μM. Two sublines were generated and designated FR1 (FTS-resistant1) and FR2 HCT-116. As shown in Fig 1A, FR1 and FR2 HCT-116 cells have gained partial resistance to FTS treatment compared to control HCT-116 cells. Accordingly, the IC50 of FR1 and FR2 HCT-116 cells were similar (57 ± 0.5) and significantly higher than the IC50 of the control HCT-116 cells (48 ± 0.55; Fig 1B). Moreover, FR1 and FR2 HCT-116 cells were more resistant to FTS-induced cell death, as demonstrated in Fig 1C and 1D.

An additional FTS-resistant subline was generated by culturing FR2 HCT-116 cells at further increasing concentrations of FTS for another 6 months (up to a concentration of 72.5 μM). This subline, termed FR3, exhibited an even greater IC50 (64 ± 0.71; S1 Fig).

### FTS-induced autophagy is altered in FTS-resistant cells

**Fig 2:**
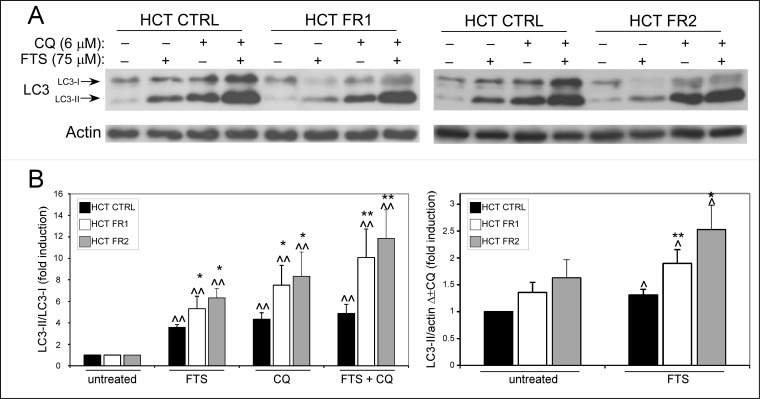
FTS resistant cells exhibit altered LC3-II/LC3-I ratio and LC3-II consumption after FTS treatment. (A) CTRL, FR1 and FR2 HCT-116 cells were treated with 75 μM FTS, with or without 6 μM chloroquine (CQ) for 48 h. The cells were then subjected to immunoblot analysis using anti-LC3 antibodies. (B) Densitometric analysis of LC3-II/LC3-I ratio is presented as fold induction over the untreated cells (left graph) and as the difference between measured values of LC3II/actin levels with or without chloroquine (right graph, Δ±CQ) (mean ± SE; *, *p* < 0.05; **, *p* < 0.01, CTRL compared to FR1 and FR2 HCT-116 cells; ^, *p* < 0.05; ^^, *p* < 0.01, FTS treated compared to untreated cells).

Previously, we have shown that FTS induces autophagy in several cancer cell lines, including HCT-116 [10,11]. FTS-induced autophagy serves as a resistance mechanism, promoting cell survival after FTS treatment [10,11]. Therefore, we next examined autophagy in the FTS resistant sublines, using LC3 protein as a marker. During autophagy, LC3-I is lipidated to form LC3-II, which binds to the autophagsomal membrane and promotes autophagy [13]. Previously, we have used the concentration range of 60–75 μM FTS in HCT-116 cells, in order to examine the effect of the treatment on cell growth, cell death and autophagy. Since the concentration of 75 μM has proven to be the most efficient in terms of autophagy induction, we decided to use it as a standard working dose in this study for the majority of the experiments. As shown in Fig 2A, FTS induced an increase in the levels of LC3-II in the control, as well as in the FR HCT-116 sublines, indicating that the treatment activates autophagy. However, in the resistant sublines, FTS also induced a reduction in LC3-I levels. These results may indicate that FTS induces a more efficient LC3-I lipidation in the FR HCT-116 cells compared to the control cells. In fact, in the presence of FTS, the LC3-II/LC3-I ratio was significantly greater in the resistant cells compared to the control cells (Fig 2B).

It should be noted that autophagy is a dynamic process, in which LC3-II formation is followed by its degradation in the lysosome. For this reason, we measured the autophagic flux using an autophagosome-lysosome fusion inhibitor, chloroquine [13]. Hence, we examined the effect of FTS and chloroquine co-treatment on LC3 levels. As shown in Fig 2A, following the combined treatment, there was no difference in the levels of LC3-II in the FR HCT-116 cells compared to the control cells; however, LC3-I levels were still lower in the FR HCT-116 cells compared to the control cells. Likewise, LC3-II/LC3-I ratio was significantly higher in the FR HCT-116 cells (Fig 2B), indicating that the lipidation step remained more efficient also in the presence of chloroquine. Next, the differences between LC3-II levels following FTS treatment alone and those following combined treatment with chloroquine were measured (Δ±CQ). Since chloroquine blocks lysosomal degradation, these differences reflect the amount of LC3-II consumed during autophagy. As demonstrated in Fig 2A and 2B, the differences were significantly greater in the FR HCT-116 cells compared to the control cells, suggesting that the process of cargo degradation had become more effective.

**Fig 3:**
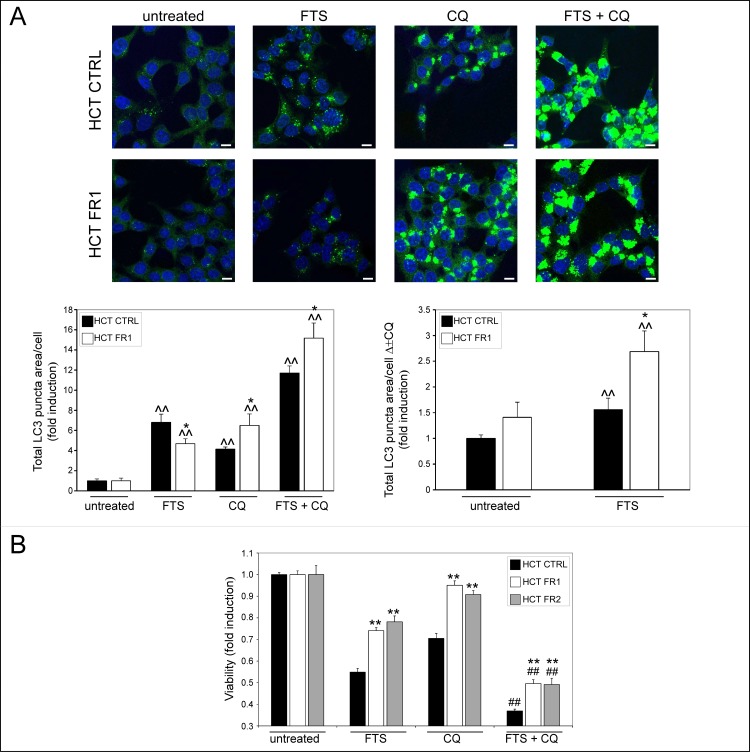
FTS resistant cells exhibit altered autophagosome consumption and increased viability following FTS and chloroquine co-treatment. (A) CTRL and FR1 HCT-116 cells were treated with 75 μM FTS, with or without 6 μM chloroquine (CQ) for 48 h. The cells were then fixed and stained with anti-LC3 antibodies and Hoechst 33258. Cells were microscopically analyzed. *Upper panels*, representative results are shown (×63 magnification; scale bars, 10 micrometer). *Lower panels*, the total area of LC3 puncta (LC3 dots) per cell was calculated using ImageJ, and is presented as fold induction over the untreated cells (left graph) or as the difference between measured values with or without chloroquine (right graph, Δ±CQ) (mean ± SE; *, *p* < 0.05; **, *p* < 0.01, CTRL compared to FR1 HCT-116 cells; ^, 0.05 < 0.05; ^^, *p* < 0.01, FTS treated compared to untreated cells). (B) CTRL, FR1 and FR2 HCT-116 cells were treated with 55 μM FTS with or without 6 μM chloroquine for 4 days. Cell viability was then assessed using the methylene blue staining assay (mean ± SE; **, *p* < 0.01, CTRL compared to FR1 and FR2 HCT-116 cells; ##, *p* < 0.01, FTS combined with chloroquine compared to each treatment alone).

To confirm the results obtained by immunoblotting, we have also used immunostaining with anti-LC3 antibodies to examine the autophagosomes content, following treatment with FTS, chloroquine or both. As shown in Fig 3A, FTS induced an increase in the total area of autophagosomes (LC3 puncta) per cell after 48 h treatment; however, this increase was greater in the control compared to the FR HCT-116 cells. The addition of chloroquine led to a further increase in the autophagosomes area, probably as a result of late-stage autophagy blockage. Under these conditions, however, the total area of autophagosomes per cell in the control HCT-116 cells was slightly lower compared to that of the FR HCT-16 cells. Accordingly, the difference between autophagosome area after FTS treatment alone and that following combined treatment with chloroquine (Δ±CQ), was significantly higher in the FR HCT-116 cell compared to the control cells (Fig 3A). Since these differences reflect autophagosome consumption following FTS treatment, the results obtained using immunostaining are in accordance with the immunoblot profile.

Chloroquine was shown to enhance the effect of FTS on cancer cell growth inhibition, death and tumorigenicity [11]. This combined effect of FTS and chloroquine on cell viability was also evident in the FR HCT-116 sublines (Fig 3B); however, the FR HCT-116 cells exhibited a partial resistance to the co-treatment compared to the control HCT-116 cells. Surprisingly, the FR HCT-116 sublines were also resistant to chloroquine treatment alone. These findings could be explained by the increased LC3-II/LC3-I ratio in the FR compared to control HCT-116 cells, observed following chloroquine, FTS or both treatments (Fig 2A and 2B). Taken together, the results imply that FTS-induced autophagy has become more efficient in the FR HCT-116 sublines, which may affect their resistance to FTS treatment.

### FTS-induced cleavage of p62 is reduced in FTS-resistant cells

**Fig 4:**
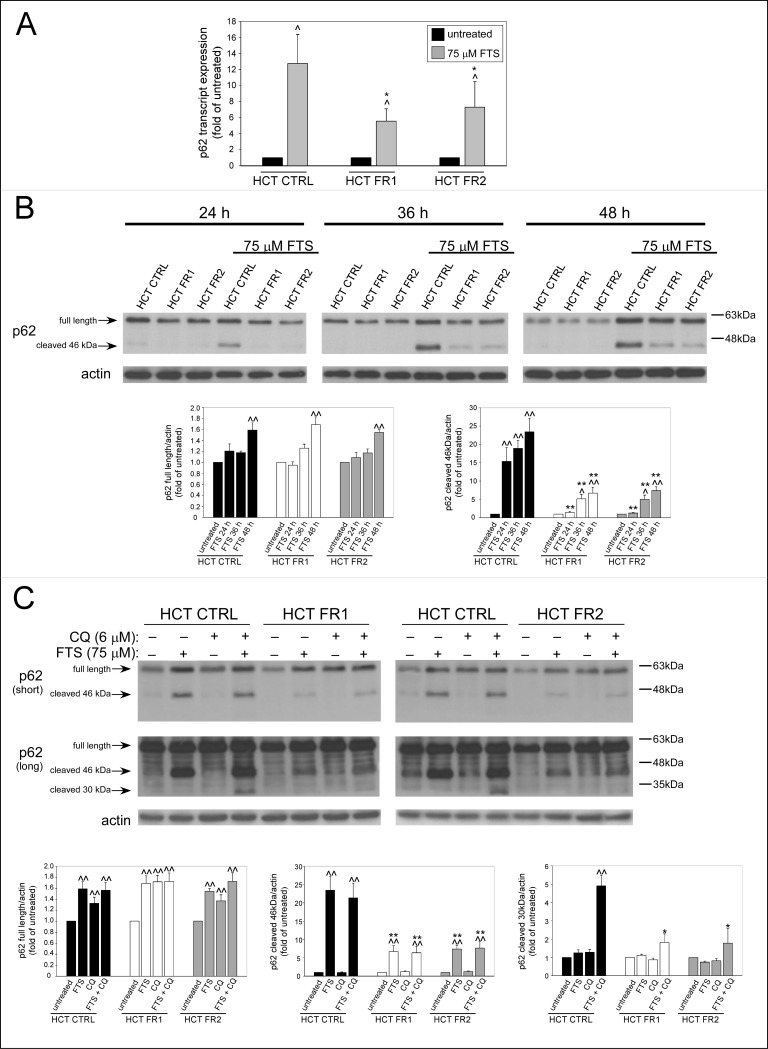
FTS-induced p62 cleavage is reduced in FTS-resistant cells. (A) CTRL, FR1 and FR2 HCT-116 sublines were treated with 75 μM FTS for 48 h and then subjected to qRT-PCR analysis of p62 transcript expression. Results are presented as fold induction over the untreated cells (mean ± SE; *, *p* < 0.05; CTRL compared to FR1 and FR2 HCT-116 cells; ^, *p* < 0.05, FTS treated compared to untreated cells). (B) CTRL, FR1 and FR2 HCT-116 sublines were treated with 75 μM FTS for the indicated time, or (C) 75 μM FTS combined with 6 μM chloroquine (CQ) for 48 h. Cell lysates were then subjected to immunoblot analysis using anti-p62 antibodies. *Upper panels*, representative results, short and long exposures are shown for FTS and CQ co-treatment blots. *Lower panels*, densitometric analysis of the results is presented as fold induction over the untreated cells (mean ± SE; *, *p* < 0.05; **, *p* < 0.01, CTRL compared to FR1 and FR2 HCT-116 cells; ^, *p* < 0.05; ^^, *p* < 0.01, FTS treated compared to untreated cells).

The ubiquitin-binding protein, p62 (also known as SQSTM1), mediates the degradation of ubiquitinated proteins and mitochondria through autophagy [14,15], by binding LC3, and recruiting the cargo to the autophagosome. This process is followed by p62 degradation in the lysosome, leading to decreased p62 levels following autophagy induction. On the other hand, persistent pro-autophagic stimuli can induce transcription-dependent synthesis of p62, resulting in elevated levels of the protein [16]. Previously, we have found that 48–72 h FTS treatment triggers *de novo* synthesis of p62 [11]. Accordingly, here we found that FTS increased the levels of p62 mRNA, and that this increase was significantly higher in the control HCT-116 cells compared to the FR cells (Fig 4A). At the protein level, FTS also induced synthesis of p62; however, there was no significant difference between the FR and the control HCT-116 cells (Fig 4B). These differences between the mRNA and the protein level can be explained by a possible cleavage of p62, which is greater in the control HCT-116 cells. Indeed, cells treated with FTS expressed a 46 kDa band, which might represent a cleavage product of p62 (Fig 4B). The alleged cleavage product appeared after a 24 h treatment with FTS, and became more prominent as the treatment continued. In addition, the levels of this 46 kDa product were significantly higher in the control HCT-116 cells compared to the FR cells, which led us to suspect that it is related to cell death. In accordance with this possibility, it was reported that p62 is cleaved by the pro-apoptotic proteins caspase 6, caspase 8 and calpain, to yield a 46 kDa product [17]. Furthermore, another apoptosis-related p62 cleavage product of 30 kDa was reported. This smaller product was detected only following co-treatment with FTS and chloroquine, but not after treatment with FTS alone (Fig 4C, long exposure), which might indicate an enhancement of apoptosis under these conditions. Consistent with that assumption, we have previously demonstrated that chloroquine enhances FTS-induced apoptosis [11]. Accordingly, the levels of the 30 kDa cleavage product were significantly higher in the control HCT-116 cells compared to those in the FR cells. Further experiments should be performed in order to verify the involvement of caspase 6 and caspase 8.

**Fig 5:**
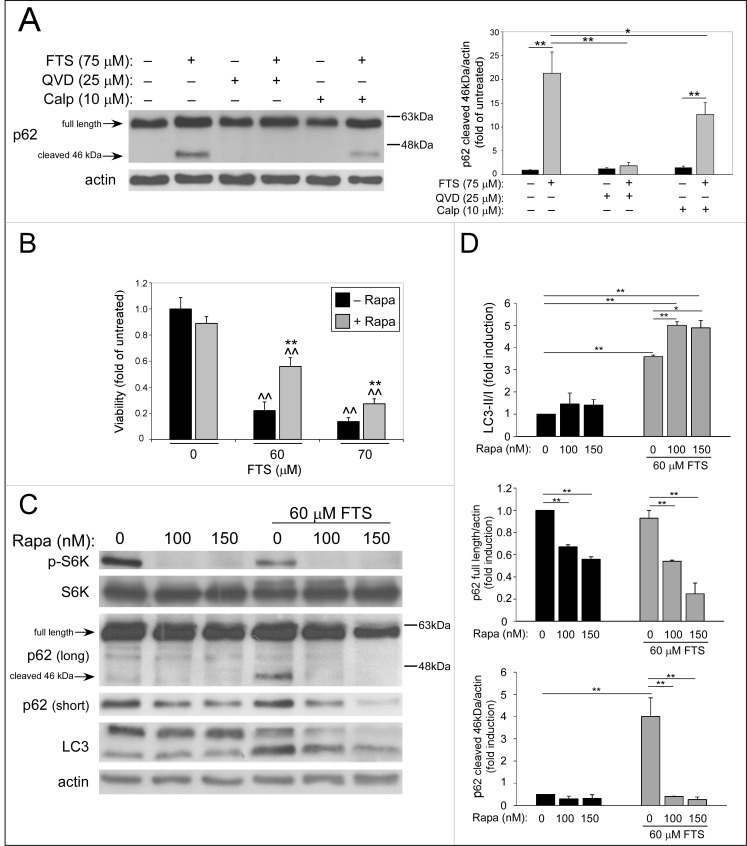
QVD, Calpeptin and rapamycin treatments inhibit the FTS-induced p62 cleavage. (A) CTRL HCT-116 cells were treated with 75 μM FTS for 48 h, in the presence or in the absence of 25 μM QVD-OPH or 10 μM calpeptin (Calp) and then subjected to immunoblot analysis using anti-p62 antibodies. *Left panel*, representative blot is shown. *Right panel*, densitometric analysis of the results is presented as fold induction over the untreated cells (mean ± SE; *, *p* < 0.05; **, *p* < 0.01). (B) CTRL HCT-116 cells were treated with FTS at the indicated concentrations for 3 days, with or without 100 nM rapamycin (rapa). Cell viability was then assessed using the methylene blue staining assay (mean ± SE; **, *p* < 0.01, FTS and rapamycin co-treatment compared to FTS treatment alone; ^^, *p* < 0.01, treated compared to untreated cells). (C) CTRL HCT-116 cells were treated with 60 μM FTS, with or without rapamycin at the indicated concentrations for 48 h. The cells were then subjected to immunoblot analysis using anti-LC3, anti-p62, anti-phospho S6K (p-S6K) and S6K antibodies. (D) Densitometric analysis of LC3-II/LC3-I ratio, full length p62 and cleaved p62 is presented as fold induction over the untreated cells (mean ± SE; *, *p* < 0.05; **, *p* < 0.01).

To confirm that FTS-induced cleavage of p62 results from enhanced apoptosis, control HCT-116 cells were treated with FTS in the presence of either QVD-OPH (pan-caspase inhibitor) or calpeptin (calpain inhibitor) (Fig 5A). The results obtained show that QVD-OPH completely abolished the cleavage of p62, while calpeptin had a partial inhibitory effect on p62 cleavage. Thus, it appears that FTS induces apoptosis-dependent cleavage of p62, which is significantly greater in the control HCT-116 cells than in the FTS-resistant cells.

To further strengthen the notion that FTS-induced cleavage of p62 is related to the induction of apoptosis, we tested the effect of rapamycin on p62 cleavage. Rapamycin, an inhibitor of mTOR, is a known inducer of autophagy. As judged by the levels of full-length p62 and LC3-II/LC3-I ratio, rapamycin enhanced the pro-autophagic effect of FTS in the control HCT-116 cells (Fig 5C and 5D). In addition, rapamycin had partially reversed the inhibitory effect of FTS on cell viability (Fig 5B), probably as a result of autophagy enhancement. As expected, rapamycin also abolished the cleavage of p62 (Fig 5C and 5D), which might indicate that the protective effect of rapamycin originates from attenuation of FTS-induced apoptosis.

FTS was also shown to induce protective autophagy in the human pancreatic cancer cell line, Panc-1 [11]. In addition, chloroquine enhanced FTS-induced apoptosis in these cells. Therefore, we tested whether FTS will have a similar effect on p62 cleavage in Panc-1 cells (S2 Fig). We found that FTS induced cleavage of p62 to yield a 46 kDa product. In the presence of chloroquine a 30 kDa fragment was observed. Thus, FTS-induced cleavage of p62 also occurs in cancer cell lines other than HCT-116.

### FTS-resistant cells exhibit reduced apoptotic features

**Fig 6:**
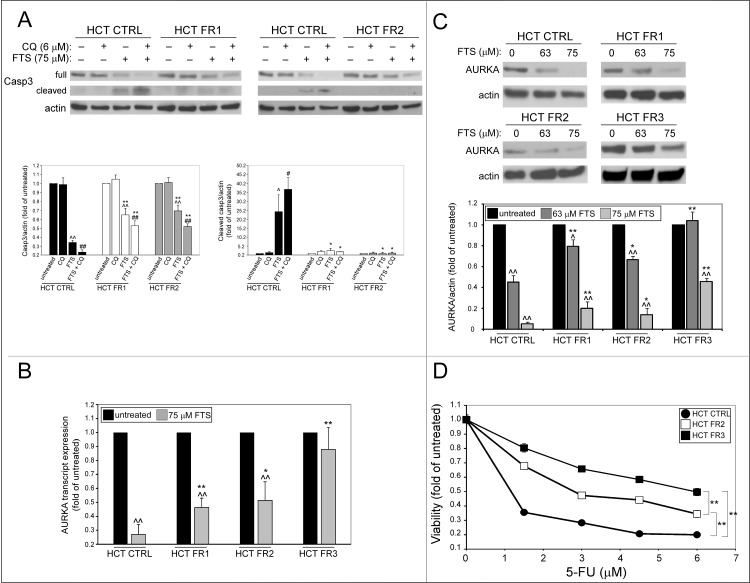
FTS-resistant cells exhibit reduced apoptosis. (A) CTRL, FR1 and FR2 HCT-116 sublines were treated with 75 μM FTS, with or without 6 μM chloroquine (CQ) for 48 h and then subjected to immunoblot analysis using anti-caspase 3 (Casp3) antibodies. *Upper panel*, representative results are shown. *Lower panel*, Densitometric analysis of the results is presented as fold induction over the untreated cells (mean ± SE; **, *p* < 0.01, CTRL compared to FR1 and FR2 HCT-116 cells; ^^, *p* < 0.01, FTS treated compared to untreated cells; ##, FTS and CQ co-treatment compared to each treatment alone). (B) CTRL, FR1, FR2 and FR3 HCT-116 sublines were treated with FTS at the indicated concentrations for 48 h and then subjected to qRT-PCR analysis of AURKA transcript expression or (C) immunoblot analysis using anti-AURKA antibodies. Results are presented as fold induction over the untreated cells (mean ± SE; *, *p* < 0.05; **, *p* < 0.01, CTRL compared to HCT FR1, FR2 and FR3 HCT-116 cells; ^, *p* < 0.05; ^^, *p* < 0.01, FTS treated compared to untreated cells). (D) CTRL, FR2 and FR3 HCT-116 sublines were treated with increasing concentrations of 5-fluorouracil (5-FU) for 3 days. Cell viability was then assessed using the methylene blue staining assay (mean ± SE; **, *p* < 0.01).

Having demonstrated that the FR HCT-116 sublines are less prone to FTS-induced p62 cleavage, and that this cleavage may be related to apoptosis, we next examined other apoptotic characteristics in these cells. Fig 6A shows that FTS induced a decrease in full-length caspase 3 expression, and, concomitantly, an increase in cleaved caspase 3, which was enhanced by chloroquine, and is indicative of apoptosis. Nevertheless, the cleavage of caspase 3 following FTS treatment, with or without chloroquine, is greater in the control cells compared to the FR HCT-116 cells.

Next, we examined the levels of the tumor promoter aurora kinase A (AURKA). This protein serves as a target for several anti-cancer agents, and its downregulation leads to apoptosis in various cancer cell lines [18–21]. It was previously shown that in A549 human lung cancer cells, FTS treatment decreases AURKA mRNA levels, and inhibits its assembly at the spindle poles [22]. We found that FTS induced a dramatic decrease of AURKA mRNA and protein levels in the control HCT-116 subline (Fig 6B and 6C). Although this decrease was also evident in the FR1 and FR2 HCT-116 cells, these sublines still expressed significantly higher levels of AURKA mRNA and protein compared to the control cells; moreover, in the FR3 HCT-116 subline, FTS had no significant effect on AURKA mRNA levels and a less pronounced effect on its protein levels, compared to the other sublines (Fig 6B and 6C). Collectively, the results indicate that the FR HCT-116 sublines have developed resistance to FTS-induced apoptosis.

Next, we examined the possibility that the FR cells have developed a general resistance mechanism that allows them to tolerate other pro-apoptotic, anti-cancer, treatments. Therefore, we employed 5-fluorouracil (5-FU), a conventional treatment for colon cancer, which is known to induce apoptosis [23]. As shown in Fig 6B, the FR HCT-116 sublines have indeed gained partial resistance to 5-FU treatment, and the subline which exhibited the highest IC50 toward FTS (FR3) also showed the highest resistance to 5-FU treatment.

### FTS-resistant cells exhibit changes in proliferation/survival signaling pathways following FTS treatment

**Fig 7:**
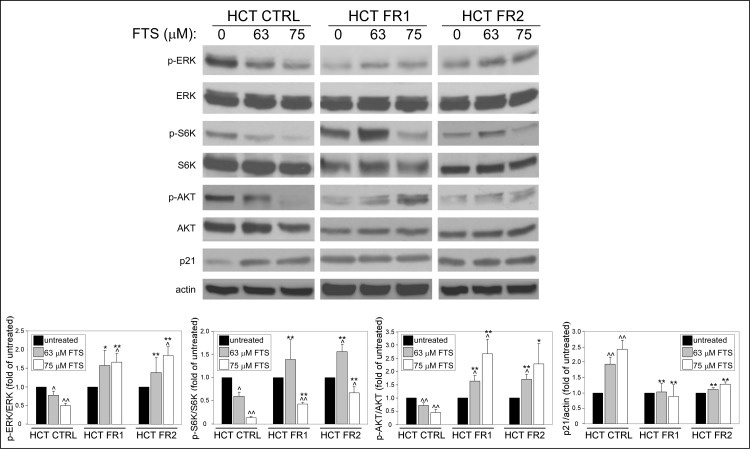
FTS-resistant cells exhibit changes in proliferation/survival signaling pathways following FTS-treatments. CTRL, FR1 and FR2 HCT-116 sublines were treated with FTS at the indicated concentrations for 24 h and then subjected to immunoblot analysis using anti-phospho-ERK, anti-phospho-S6K, anti-phospho-AKT and anti-p21 antibodies. Results are presented as fold induction over the untreated cells (mean ± SE; *, *p* < 0.05; **, *p* < 0.01, CTRL compared to FR1 and FR2 HCT-116 cells; ^, *p* < 0.05; ^^, *p* < 0.01, FTS treated compared to untreated cells).

The findings, which suggest resistance to apoptosis in the FR HCT-116 sublines, have led us to examine the effect of FTS on major signaling pathways known to be affected by Ras activation. We analyzed pathways that regulate cell cycle progression/cell proliferation (ERK), cell growth (S6K) and cell survival (AKT) 24 h following FTS treatment [1] (Fig 7). In the control HCT-116 cells, 63 and 75 μM FTS treatments induced a decrease in the levels of phosphorylated ERK and AKT (p-ERK and p-AKT, respectively). In contrast, at the same concentrations, FTS induced an increase in the levels of p-AKT and p-ERK in the FR HCT-116 cells. Similarly, FTS reduced the levels of p-S6K in the control HCT-116 cells; however, in the FR HCT-116 cells, 63 μM FTS had led to an increase in p-S6K levels while only at 75 μM FTS, p-S6K levels decreased. Nevertheless, under these conditions, p-S6K levels remained significantly higher in the FR HCT-116 cells compared to the control cells. Similar results were obtained following transfection with a dominant negative (DN) H-Ras 17N mutant (S3 Fig.), which was previously found to inhibit the downstream signaling activity of all Ras isoforms [24]. Taken together, the results suggest that in the control HCT-116 cells, cell proliferation, growth and survival are suppressed by FTS, possibly through its effect on Ras signaling. On the contrary, in the FR HCT-116 cells, these processes are activated following FTS treatment, perhaps as a compensation mechanism. Next, we examined the levels of p21 protein, which negatively regulates cell cycle progression, and whose expression was shown to increase following FTS treatment [25]. While FTS enhanced the expression of p21 in the control HCT-116 cells, it had no effect on p21 levels in the FR cells, indicating that the treatment did not affect cell cycle progression in the FR HCT-116 sublines (Fig 7). Here again, a similar effect was visible following transfection with DN-Ras (S3 Fig).

### FTS-resistant HCT-116 cells preserve their resistance characteristics after prolonged FTS withdrawal

**Fig 8:**
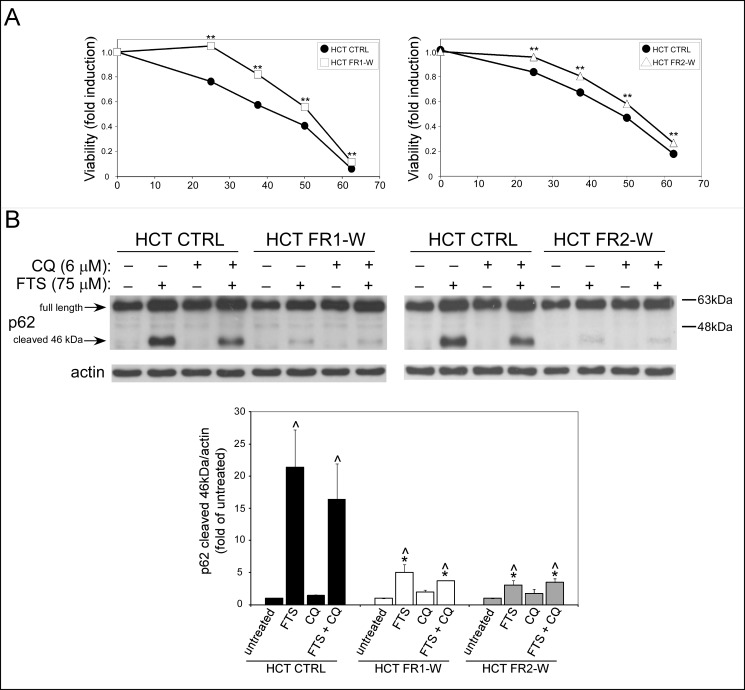
The FTS–resistant cells preserve the apoptosis resistance characteristics after one month of FTS withdrawal. FR1 and FR2 HCT-116 sublines were grown without FTS for 1 month (FR1-W and FR2-W). These cells, as well as CTRL HCT-116 cells, were then treated with increasing concentrations of FTS for 5 days after. Cell viability was assessed using the methylene blue staining assay (mean ± SE; **, p < 0.01, CTRL compared to HCT-116 FR1-W and FR2-W HCT-116 cells). (B) CTRL, FR1-W and FR2-W HCT-116 cells were treated with 75 μM FTS, with or without 6 μM chloroquine (CQ) and then subjected to immunoblot analysis using anti-p62 antibodies. Upper panel, representative blot is shown. Lower panel, densitometric analysis of the results is presented as fold induction over the untreated cells (mean ± SE; *, *p* < 0.05, CTRL compared to FR1-W and FR2-W HCT-116 cells; ^, *p* < 0.05, FTS treated compared to untreated cells).

To examine whether FTS-resistance is transient or stable, we withdrew the FR HCT-116 cells from FTS treatment for one month, and tested cell viability following 5 days treatment with FTS at a range of concentrations (Fig 8A). Compared to the control-HCT-116 cells, the FR HCT-116 cells preserved their resistance toward FTS. Moreover, the FR HCT-116 cells have also retained the low levels of cleaved p62 compared to the control cells, following treatment with FTS (Fig 8B). These findings suggest that the mechanisms involved in FTS-resistance are relatively stable.

## Discussion

Drug resistance in cancer cells poses a major barrier for tumor eradication, and for further designing of novel anti-cancer drugs. Numerous anti-cancer agents were shown to induce resistance in tumor cells following continuous treatment, *in vitro* and *in vivo*, as well as in human subjects [26]. In this study, we examined the possibility that long exposure to the Ras inhibitor, FTS, would lead to treatment resistance. Resistance to FTS following 6 months treatment was tested in the human colon cancer cell line HCT-116. We found that, indeed, the cells have developed resistance to FTS-induced growth inhibition and cell death.

Further characterization of the FTS-resistant cells revealed that their autophagy has become more efficient, as judged by the levels of LC3-I conversion into LC3-II and LC3 puncta. Another feature of the, presumably, more efficient autophagy in the FTS-resistant cells is reflected by the increased rates of cargo degradation following treatment (enhanced autophagic flux), compared to non-resistant cells, as reflected by both immunoblotting and immunostaining of endogenous LC3. This change might result from an enhanced autophagosome-lysosome fusion, an increased lysosomal activity or an increase in lysosomal content in the FTS-resistant cells. Previously, we have demonstrated that autophagy has a protective role in cancer cells treated with FTS [10,11]. Thus, the enhanced autophagy observed in the FTS-resistant cells might represent an adaptation mechanism to FTS. The results also suggest that in the non-resistant cells, autophagy does not function at its full capacity following the treatment, compared to the resistant cells. Indeed, activation of autophagy with rapamycin seems to enhance the conversion of LC3-I into LC3-II, to augment cargo degradation, and to reverse the inhibitory effect of FTS on cell viability.

The p62 protein is involved in cargo selection during autophagy. In addition, other functions were attributed to p62, some of which are related to apoptosis [27]. In the present study, we found that FTS induced cleavage of p62 in HCT-116 cells, as well as in Panc-1 cells, to yield a 46 kDa product, and that at the addition of chloroquine, a 30 kDa product was also generated. This cleavage was inhibited by QVD-OPH and calpeptin, as well as by rapamycin. Cleavage of p62 into a 46 kDa fragment was previously reported to be caspase 6- and 8-dependent, while the 30 kDa fragment depends on caspase 6 activity. Calpain was also reported to contribute to p62 cleavage, generating multiple cleavage products [17]. Thus, FTS-induced cleavage of p62 appears to be related to apoptosis induction. We have previously shown that chloroquine enhances FTS-induced apoptosis in Panc-1 and HCT-116 cells [11]. Given the fact that the 30 kDa cleavage product was detected only following combined FTS and chloroquine treatment, but not after FTS treatment alone, it may be related to the occurrence of enhanced apoptosis under these conditions. The cleavage of p62 was more prominent in non-resistant HCT-116 cells compared with FTS-resistant cells, indicating that the resistant cells exhibit reduced apoptosis following treatment. This assumption is further supported by the fact that FTS, alone and with chloroquine, had less effect on caspase 3 cleavage in the FTS-resistant HCT-116 cells, compared with the non-resistant cells. The low levels of cleaved p62 generated in the FTS-resistant cells might be linked to their resistance mechanism, since cleaved p62 was shown to inhibit autophagy, and it may possess cytotoxic activity [28,29]. Therefore, it is plausible that the reduced cleavage of p62 in the FTS-resistant cells allows them to exhibit a more effective autophagy, resulting in a higher resistance to the treatment.

In the FTS-resistant HCT-116 cells, other proteins were affected. One such protein is AURKA, which is involved in spindle assembly and cell division [18]. FTS treatment reduced the levels of AURKA mRNA and protein; however, this effect was more profound in the non-resistant HCT-116 cells compared to the resistant cells. This result pinpoints AURKA as part of an additional mechanism responsible for FTS resistance. In fact, AURKA expression was shown to potentiate Ras-induced transformation, and it correlates with colon cancer progression [18]. Moreover, AURKA inhibitors were found to be highly effective against the growth of HCT-116 cells and xenograft tumors. In accordance with these results we have found changes in signaling pathways related to cell growth (S6K, a substrate of mTOR), survival (AKT) and proliferation (Erk). FTS inhibits these pathways in the non-resistant HCT-116 cells; however, in the FTS-resistant cells, an opposite effect occurs, which may indicate that the alterations in these signaling pathways allow the cells to better cope with the treatment. Moreover, while FTS induced an increase in p21 in the non-resistant HCT-116 cells, which is indicative of cell cycle arrest, it did not affect p21 levels in the FTS-resistant cells, further showing an altered response to the treatment. It was shown that HCT-116 cells harbor a heterozygous activation mutation in PI3K [30]. Yet, intriguingly, it appears that inhibition of Ras, which occurs upstream of PI3K, by both FTS and the DN-Ras, suppressed PI3K/Akt/mTOR signaling in the non-resistant HCT-116. As for the Erk pathway, it was shown that it can activate autophagy in several cancer cell lines, including colon cancer cells [31]; therefore, the activation of this pathway by FTS in the resistant cells might explain their enhanced autophagy compared with the non-resistant cells following FTS treatment.

To summarize, we found that HCT-116 human colon cancer cells can develop resistance to the Ras inhibitor FTS, following prolonged treatment. Some of the FTS-resistance characteristics were more prominent in HCT-116 cells that were exposed to higher concentrations of FTS during the continuous treatment period (FR3 HCT-116 cells). Hence, the degree of resistance seems to depend upon FTS concentration. It also appears that the acquired resistance to FTS is quite stable, since it was still evident one month after FTS withdrawal. Several features appear to be altered in the FTS-resistant HCT-116 cells, some of which might account for FTS-resistance. In addition, the mechanisms involved in FTS-resistance might represent a more general phenomenon, since the FTS-resistant HCT-116 cells were also less affected by treatment with 5-FU. The suggested resistance mechanisms described here might assist in understanding acquired resistance mechanisms for anticancer therapy consisting of anti-Ras agents in combinations with other inhibitors.

## Supporting Information

S1 Fig Effect of FTS on cell viability in CTRL and FTS-resistant HCT-116 (FR3) sublines. CTRL and FR3 HCT-116 sublines, were treated with increasing concentrations of FTS for 5 days. Cell viability was then assessed using the methylene blue staining assay (A) and IC50 values were calculated (B) as described in materials and methods (**, *p* < 0.01, CTRL compared to FR3 HCT-116 cells).
(TIF)

S2 Fig Effect of FTS and chloroquine on p62 cleavage in Panc-1 cells. Panc-1 cells were treated with FTS for the indicated concentrations and times (A) or for 96 h in combination with 15 μM chloroquine (CQ) (B). The cells were then subjected to immunoblot analysis using anti-p62 antibodies.
(TIF)

S3 Fig FTS-resistant cells exhibit changes in proliferation/survival signaling pathways following transfection with a dominant negative (DN) Ras variant. CTRL, FR1 and FR2 HCT-116 sublines were transfected with either GFP or GFP-DN-Ras for 48 h and subjected to immunoblot analysis using anti-phospho-S6K, anti-phospho-AKT and anti-p21 antibodies. Numbers below bands indicate fold induction of total protein /actin levels. The results shown are of a representative experiment.
(TIF)

## Abbreviations

5-FU: 5-fluorouracil
AURKA: aurora kinase A
CQ: chloroquine
FR: FTS-resistant
FTS: farnesylthiosalicylic acid
LC3: microtubule associated protein light chain 3
Rapa: rapamycin
S6K: ribosomal protein S6 kinase
