# Supplementary figures and images for: Continuous treatment with FTS confers resistance to apoptosis and affects autophagy
Source: PLoS One. 2017 Feb 2;12(2):e0171351. doi: 10.1371/journal.pone.0171351 (PMC5289601; doi:10.1371/journal.pone.0171351)

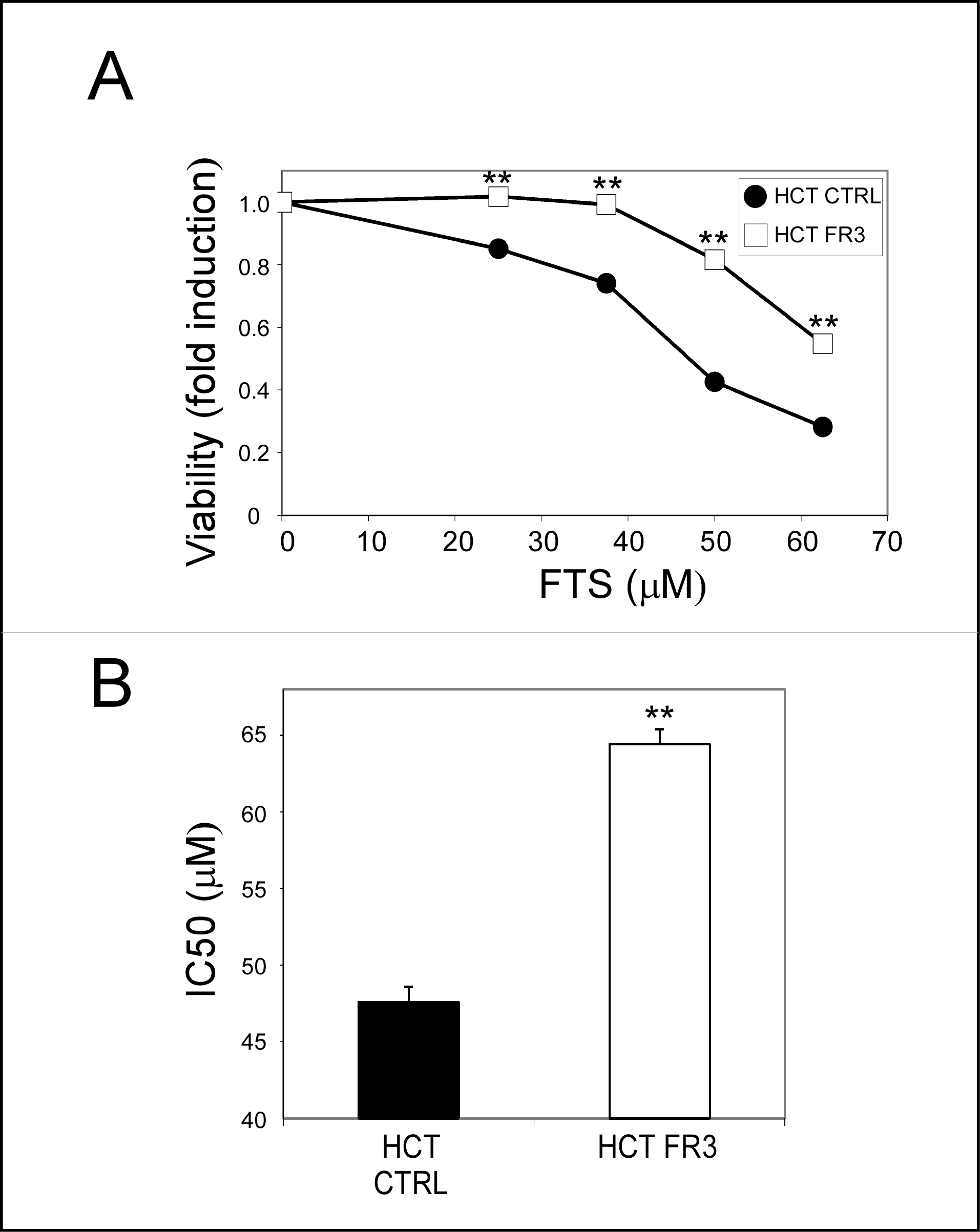

Supplement: S1 Fig — CTRL and FR3 HCT-116 sublines, were treated with increasing concentrations of FTS for 5 days. Cell viability was then assessed using the methylene blue staining assay (A) and IC50 values were calculated (B) as described in materials and methods (**, p < 0.01, CTRL compared to FR3 HCT-116 cells). (TIF) [file pone.0171351.s001.tif]

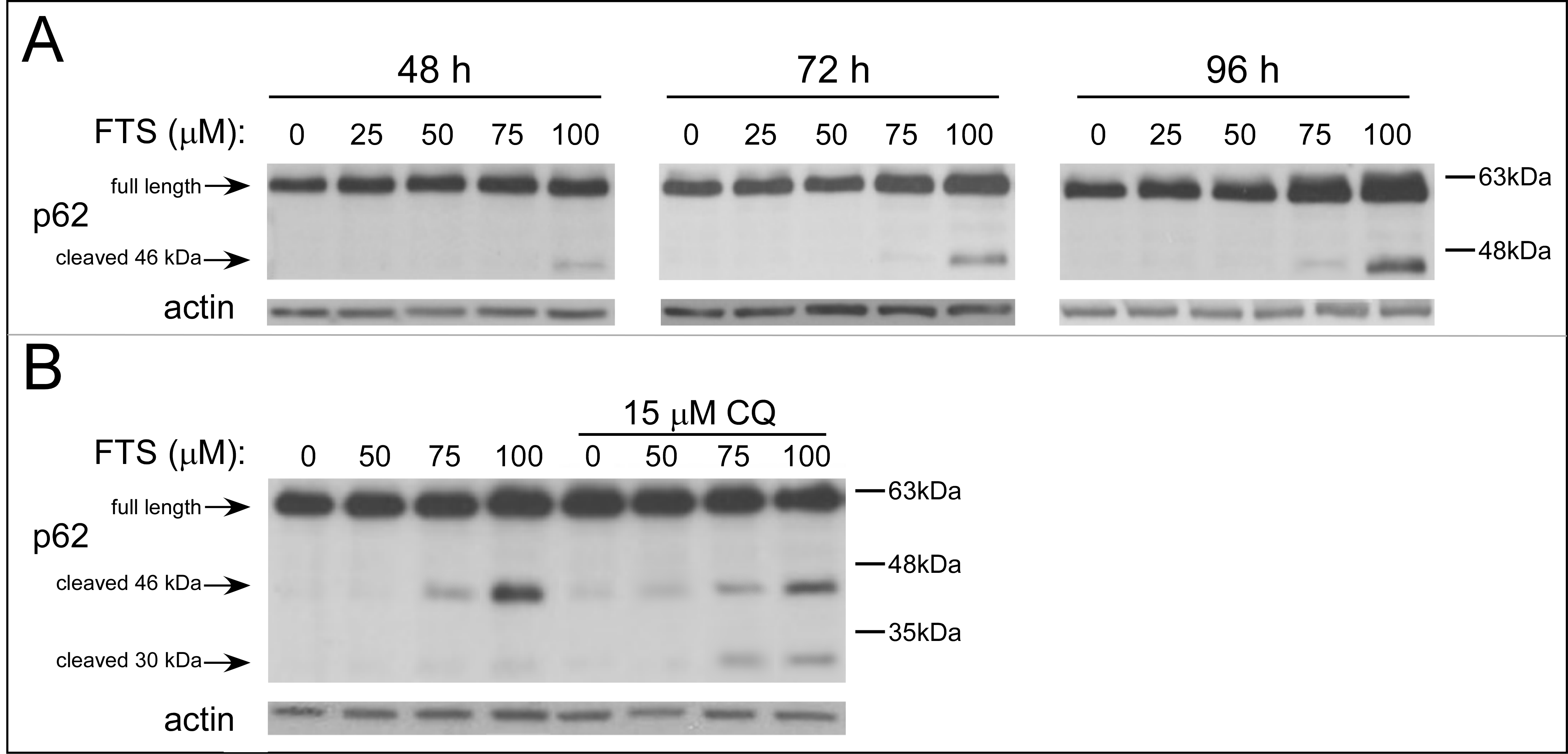

Supplement: S2 Fig — Panc-1 cells were treated with FTS for the indicated concentrations and times (A) or for 96 h in combination with 15 μM chloroquine (CQ) (B). The cells were then subjected to immunoblot analysis using anti-p62 antibodies. (TIF) [file pone.0171351.s002.tif]

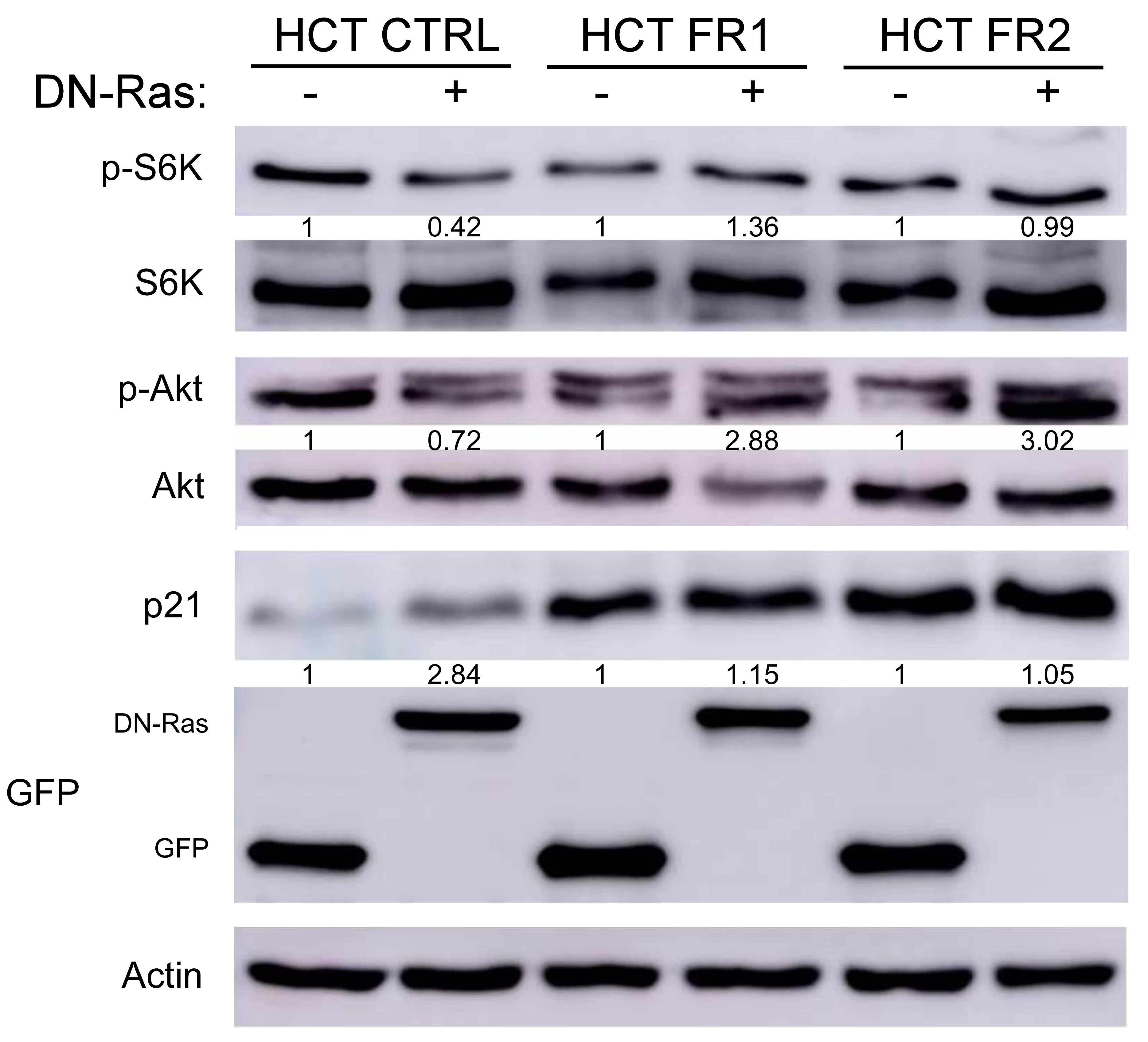

Supplement: S3 Fig — CTRL, FR1 and FR2 HCT-116 sublines were transfected with either GFP or GFP-DN-Ras for 48 h and subjected to immunoblot analysis using anti-phospho-S6K, anti-phospho-AKT and anti-p21 antibodies. Numbers below bands indicate fold induction of total protein /actin levels. The results shown are of a representative experiment. (TIF) [file pone.0171351.s003.tif]
